# Assessing availability of scientific journals, databases, and health library services in Canadian health ministries: a cross-sectional study

**DOI:** 10.1186/1748-5908-8-34

**Published:** 2013-03-21

**Authors:** Grégory Léon, Mathieu Ouimet, John N Lavis, Jeremy Grimshaw, Marie-Pierre Gagnon

**Affiliations:** 1 Centre Hospitalier Universitaire de Québec Research Centre, Hôpital St-François D’Assise, Quebec City, QC, Canada; 2 Department of Political Science, Université Laval, Quebec City, QC, Canada; 3 McMaster Health Forum, McMaster University, Hamilton, ON, Canada; 4 Centre for Health Economics and Policy Analysis, McMaster University, Hamilton, ON, Canada; 5 Department of Clinical Epidemiology and Biostatistics, McMaster University, Hamilton, ON, Canada; 6 Department of Political Science, McMaster University, Hamilton, ON, Canada; 7 Ottawa Hospital Research Institute, Ottawa, ON, Canada; 8 Department of Medicine, University of Ottawa, Ottawa, ON, Canada; 9 Faculty of Nursing Science, Université Laval, Quebec City, QC, Canada

**Keywords:** Health care, Information science, Library science, Knowledge transfer, Research evidence

## Abstract

**Background:**

Evidence-informed health policymaking logically depends on timely access to research evidence. To our knowledge, despite the substantial political and societal pressure to enhance the use of the best available research evidence in public health policy and program decision making, there is no study addressing availability of peer-reviewed research in Canadian health ministries.

**Objectives:**

To assess availability of (1) a purposive sample of high-ranking scientific journals, (2) bibliographic databases, and (3) health library services in the fourteen Canadian health ministries.

**Methods:**

From May to October 2011, we conducted a cross-sectional survey among librarians employed by Canadian health ministries to collect information relative to availability of scientific journals, bibliographic databases, and health library services. Availability of scientific journals in each ministry was determined using a sample of 48 journals selected from the 2009 Journal Citation Reports (Sciences and Social Sciences Editions). Selection criteria were: relevance for health policy based on scope note information about subject categories and journal popularity based on impact factors.

**Results:**

We found that the majority of Canadian health ministries did not have subscription access to key journals and relied heavily on interlibrary loans. Overall, based on a sample of high-ranking scientific journals, availability of journals through interlibrary loans, online and print-only subscriptions was estimated at 63%, 28% and 3%, respectively. Health Canada had a 2.3-fold higher number of journal subscriptions than that of the provincial ministries’ average. Most of the organisations provided access to numerous discipline-specific and multidisciplinary databases. Many organisations provided access to the library resources described through library partnerships or consortia. No professionally led health library environment was found in four out of fourteen Canadian health ministries (i.e. Manitoba Health, Northwest Territories Department of Health and Social Services, Nunavut Department of Health and Social Services and Yukon Department of Health and Social Services).

**Conclusions:**

There is inequity in availability of peer-reviewed research in the fourteen Canadian health ministries. This inequity could present a problem, as each province and territory is responsible for formulating and implementing evidence-informed health policies and services for the benefit of its population.

## Background

In Canada, the provincial and territorial health ministries are responsible for developing legislation, regulations, policies and directives to support strategic directions, and in some cases, operational decision-making for their health care systems (see [1] for more information about Canada’s health care system). Cohesion within this decentralised health system is maintained by national standards through the Canada Health Act [2]. Since the end of the 1990s, leaders of governments, such as in Canada [3,4], the United States, the United Kingdom and Australia, have emphasised the importance of developing an evidence-informed health system, with decisions made on the basis of appropriate, balanced and high-quality evidence.

Evidence-informed health policymaking is described as an approach to policy decisions that aims to ensure that decision making is well-informed by the best available research evidence among other factors (e.g. institutional constraints, interest group pressures, and citizen values) [5]. This approach was formally adapted to public health by scholars and public health practitioners about 14 years ago on the principles of evidence-based medicine [6,7]. Public health surveillance, evidence-based guidelines, review-derived products (e.g. summaries and overviews of systematic reviews, policy briefs based on systematic reviews), primary research studies and the gray literature are all relevant sources of information for public health and health system policy decision makers [8]. Public health and health promotion decision makers generally support the development of a readily accessible and easy-to-use source of research information [9-12]. However, developing information resources for health policy is a challenging process because of the range of decision-making settings and the tremendous diversity in the nature of information needed in the field of medicine, epidemiology, nursing, sociology, political science, administration, economics, law, statistics, public administration, engineering, and other disciplines [13].

Over the past ten years, studies have been conducted around the world on the relation between research evidence and policymaking to identify some of the key issues and potential solutions to promote the development and application of evidence in health care decision making [8,11,14-17]. Public health decision makers strongly indicated a need to receive research evidence that is specific to their decision-making environments and appears user-friendly in a concise format [18-20]. Several initiatives to retrieve research evidence, such as the development of web-based resources (e.g. Medline Plus, MORE EBN, Health Systems Evidence, Health-evidence.ca), have tended to focus on making information available and accessible (in a timely fashion) to decision makers in order to increase their capacity to use research [8,21-24]. Moreover, intense efforts have been expended on developing knowledge management tools and services in order to enhance the use of systematic reviews (SRs) by health policy makers and managers [25-28]. Over the past years, researchers and decision makers participated in knowledge transfer and exchange activities to increase the use of research in health policy. However, recent reviews of the literature showed that few studies pertain to the implementation and evaluation of the effectiveness of these activities [15,16].

Several factors contribute to the limited application of research evidence within program decision making and policy development [29-32]. Major barriers to evidence-informed decision making include lack of time, limited availability and access to research evidence, and limited user capacity [18,31,33-35]. A recent systematic review of the literature showed that little information is available on the extent to which research evidence is used in public health decision-making processes. Although a range of different types of research evidence was accessed, the authors of this review found that research evidence impact on policy was often indirect and competed with other influences [18].

Physical access to research evidence is a prerequisite of research utilisation by policy makers [11,17,18]. For the past four years, the Canadian Virtual Health Library has worked towards the pursuit of equal access to health information for all Canadian health professionals, including public health workers and health care system administrators [36]. However, Canada still lacks a national health information service that promotes the convergence of initiatives and resources across health system organisations [37]. National online library initiatives that enhance equal access to quality-assured health information are found in many countries, such as the United States (the National Network of Libraries of Medicine [38]), the United Kingdom (NHS Evidence [39] and the Knowledge Network [40]), Norway (the Electronic Health Library [41]), Iceland (the National and University Library [42]) and Australia (the National Library of Australia [43]).

Among the requirements for research evidence to further inform decision making, public health decision makers indicate that i) “evidence should arise from sources which are seen as unbiased (such as peer-reviewed research), authoritative and credible; and provide methodological details so rigour can be assessed” and ii) “research evidence should be made more widely available to decision makers through the use of email bulletins, public health professional organisations or clearinghouses” [18]. To our knowledge, despite the substantial political and societal pressure to enhance the use of the best available research evidence in public health policy and program decision-making, there is no study addressing availability of peer-reviewed research in Canadian health ministries. The aim of this study was three-fold: to assess availability of (1) a purposive sample of high-ranking scientific journals, (2) bibliographic databases, and (3) health library services in the fourteen Canadian health ministries.

## Methods

### Ethics

The Laval University Institutional Review Board (IRB) established that this research project did not fall within the second edition of the “Tri-Council Policy Statement. For that reason, the Laval University IRB did not require further assessment and approval. However, proper arrangements were made to assure the participants’ (informants’) anonymity in analysis, presentation and publication of the data.

### Study design, setting and participants

We used a cross-sectional survey to collect information relating to availability of a purposive sample of scholarly journals, bibliographic databases, and health library services in the following fourteen Canadian health ministries: Health Canada, British Columbia Ministry of Health Services, Alberta Health and Wellness, Saskatchewan Ministry of Health, Manitoba Health, Ontario Ministry of Health and Long-Term Care, Quebec Ministry of Health and Social Services, New Brunswick Department of Health, Nova Scotia Department of Health and Wellness, Prince Edward Island Department of Health and Wellness, Newfoundland and Labrador Department of Health and Community Services, Yukon Department of Health and Social Services, Northwest Territories Department of Health and Social Services, and Nunavut Department of Health and Social Services. Here, assessment of availability of journals, databases and library services in these organisations included the access provided through library partnerships or consortia.

Informants were: one library employee (e.g. manager, librarian, technician or records clerk) from each ministry, except for the territorial health ministries because these organisations had no department library at the time of this study. Instead, the most relevant resource persons available were two legislative librarians for the Northwest Territories Department of Health and Social Services and the Nunavut Department of Health and Social Services, and a senior policy advisor for the Yukon Department of Health and Social Services. The number of informants was purposely limited to one per organisation (n = 14), because the survey questionnaire aimed at collecting basic information at the organisational level, as opposed to the individual level (i.e., perceptions/views about health library resource and service). Also, some organisations had limited (e.g., Manitoba Health) or no (e.g., the territorial health ministries) staff assigned to health library services. The limited number of participants allowed timely response to the participants’ information request. Reminder e-mails were sent every two weeks until the survey closed.

### Data collection

The contact information of potential informants was obtained through the ministries’ websites (listed on the Health Canada website at [44]), by contacting their communication divisions or by other contacts, when necessary. Then, the informants were contacted by GL by email and informed about the project. On accepting to participate, the informants were invited to complete a survey (available as a fillable PDF form) and to send it back by email to GL. Participants were asked to take as much time as needed to answer the survey and, when necessary, to go look at the resources available and/or to contact their colleagues to verify the accuracy of their responses before sending their responses. When a health library infrastructure was not present, or when the contacted informant declined to participate, we asked for the contact information of the most relevant resource persons available until one participant was found per organisation. The only inclusion criterion was being able to answer basic organisational questions relative to the current availability of journals, databases and library services. If more than one informant per organisation was eligible, the first respondent that agreed to participate was chosen and the other(s) was (were) thanked. The survey was composed of four questions: “Q1: Does your department library have a print and/or an online subscription to the following journals?”, “Q2: Does your department library have an online catalogue? If yes, please provide the URL address”, “Q3: Does your department library have a partnership and/or is a member of a resource-sharing network? If yes, please provide a description of the network and its services”, and “Q4: Does your department library subscribe to any health or science databases? If yes, please list them all”. The first question focuses on the type of access that a health ministry might provide for a purposive sample of scientific journals (see below for information about the method we used for selecting the journals). More specifically, we collected data for the following types of access: online subscription, print-only subscription, interlibrary loan, and ‘no access’ using one of the previous means.

For the first question, a list of scientific journals sorted by subject category and then by impact factor was determined in March 2011 using the 2009 Journal Citation Reports (JCR) published online by Thomson Reuters. Based on scope note information about subject categories, eight categories from the 2009 Science Edition (i.e. “Health Care Sciences & Services”, “Medical Ethics”, “Medical Informatics”, “Medicine, General & Internal”, “Medicine, Legal”, “Medicine, Research & Experimental”, “Nursing” and “Public, Environmental & Occupational Health”) and three categories from the 2009 Social Sciences Edition (i.e. “Health Policy & Services”, “Public, Environmental & Occupational Health” and “Social Sciences, Biomedical”) of the JCR were chosen because of their relevance for health policy. Then, in order to narrow our journal sample, we used the journal impact factors as an indicator of journal popularity and selected the five top-ranked journals for each journal category. Six journal duplicates found in more than one category were removed from the sample, whereas three Canadian journals (i.e. *Canadian Medical Association Journal*, *Canadian Journal of Public Health*, and *Healthcare Policy*) and one of the most exclusive journals of systematic literature reviews for health care decision making (i.e. *Cochrane Database of Systematic Reviews*) were added to the sample. *Healthcare Policy* was the only journal in our sample that was not evaluated in the 2009 JCR. Finally, journals that publish open access (OA) articles (i.e. direct OA, hybrid OA and delayed OA) were identified. Table 1 shows the list of 53 scientific journals that were selected and included in the survey. The availability indicator taken into account in this study is the number of journals (from our sample) that are accessible using one of the following types of access: 1) online subscription (with or without an additional print subscription), 2) print-only subscription, 3) interlibrary loan, and 4) ‘no access’. The detailed results of availability by journal and ministry are presented in Additional file 1.

**Table 1:** List of 53 scientific journal titles included in the survey

**JCR**^**a **^**Edition (year)**	**Subject category**	**Journal (Information relative to access and content)**^**b**^	**IF**^**c **^**(Rank)**	**Id**^**d**^
Science (2009)	Health Care Sciences & Services	-Health Technology Assessment (OA) (SR)	6.910 (1)	1
		-Journal of Medical Internet Research (OA) (SR)	3.924 (2)	2
		-Milbank Quarterly (SR)	3.872 (3)	3
		-Health Affairs (SR)	3.582 (4)	4
		-Medical Care (SR)	3.241 (5)	5
	Medical Ethics	-American Journal of Bioethics	4.000 (1)	6
		-Ethnicity & Health (SR)	1.673 (2)	7
		-Hastings Center Report	1.539 (3)	8
		-Journal of Law, Medicine & Ethics	1.433 (4)	9
		-Developing World Bioethics (*)	1.256 (5)	10
	Medical Informatics	-Journal of the American Medical Informatics Association (*) (SR)	3.974 (1)	11
		-Journal of Medical Internet Research (OA) (SR)	3.924 (2)	D_2_
		-International Journal of Medical Informatics (SR)	3.126 (3)	12
		-Medical Decision Making (SR)	2.597 (4)	13
		-Statistical Methods in Medical Research	2.569 (5)	14
	Medicine, General & Internal	-New England Journal of Medicine (*)	47.050 (1)	15
		-Lancet (SR)	30.758 (2)	16
		-JAMA-Journal of the American Medical Association (SR)	28.899 (3)	17
		-Annals of Internal Medicine (*) (SR)	16.225 (4)	18
		-British Medical Journal (OA) (SR)	13.660 (5)	19
		-Canadian Medical Association Journal (*) (SR)	7.271 (9)	20
		-Cochrane Database of Systematic Reviews (**) (SR)	5.653 (11)	21
	Medicine, Legal	-International Journal of Legal Medicine	2.793 (1)	22
		-Forensic Science International-Genetics	2.421 (2)	23
		-Forensic Science International (SR)	2.104 (3)	24
		-Regulatory Toxicology and Pharmacology (SR)	1.798 (4)	25
		-Journal of Forensic Sciences	1.524 (5)	26
	Medicine, Research & Experimental	-Nature Medicine	27.136 (1)	27
		-Journal of Clinical Investigation (*)	15.387 (2)	28
		-Journal of Experimental Medicine (*)	14.505 (3)	29
		-Trends in Molecular Medicine	11.049 (4)	30
		-Molecular Aspects of Medicine	6.492 (5)	31
	Nursing	-Worldviews on Evidence-Based Nursing (SR)	1.944 (1)	32
		-Birth-Issues in Perinatal Care (SR)	1.919 (2)	33
		-International Journal of Nursing Studies (SR)	1.910 (3)	34
		-Oncology Nursing Forum (SR)	1.907 (4)	35
		-Cancer Nursing (SR)	1.878 (5)	36
	Public, Environmental & Occupational Health	-Epidemiologic Reviews (*) (SR)	17.500 (1)	37
		-WHO Technical Report Series (OA) (SR)	8.000 (2)	38
		-Annual Review of Public Health (SR)	7.915 (3)	39
		-Environmental Health Perspectives (OA) (SR)	6.191 (4)	40
		-American Journal of Epidemiology (*) (SR)	5.589 (5)	41
Social Sciences (2009)	Health Policy & Services	-Milbank Quarterly (SR)	3.872 (1)	D_3_
		-Health Affairs (SR)	3.582 (2)	D_4_
		-Medical Care (SR)	3.241 (3)	D_5_
		-Value in Health (SR)	3.032 (4)	42
		-Psychiatric Services (SR)	2.813 (5)	43
	Public, Environmental & Occupational Health	-Annual Review of Public Health (SR)	7.915 (1)	D_39_
		-American Journal of Public Health (SR)	4.371 (2)	44
		-Tobacco Control (SR)	3.852 (3)	45
		-Journal of Adolescent Health (SR)	3.325 (4)	46
		-Journal of Epidemiology and Community Health (SR)	3.043 (5)	47
		-Canadian Journal of Public Health (*) (SR)	1.349 (45)	48
	Social Sciences, Biomedical	-American Journal of Bioethics	4.000 (1)	D_6_
		-Evolution and Human Behavior	3.594 (2)	49
		-Aids and Behavior (SR)	3.038 (3)	50
		-Social Science & Medicine (SR)	2.710 (4)	51
		-Psycho-Oncolog (SR)	2.684 (5)	52
NA^e^	NA	-Healthcare Policy (*) (SR)	NA	53

^a^JCR, Journal Citation Report.

^b^Information collected from the journals’ websites. (OA), Open access journal: All research articles are freely available online; (*), Hybrid or delayed open access journal; (**), Free online access is made available through funded provisions in New Brunswick, Nova Scotia and Saskatchewan. (SR), Journal contains systematic reviews in present and/or past issues.

^c^IF, Impact factor and rank (based on impact factor) adapted from the 2009 JCR.

^d^Id, Identification number attributed to each journal tested in this study. D_(Id)_, Duplicated journal entry for a designated journal Id within this table.

^e^NA, Not Applicable. *Healthcare Policy* was not evaluated in the 2009 JCR.

### Data analysis

Data collected in the survey were used to generate five tables of data that describe availability of scientific journals (Tables 2 and 3, and Additional file 1), bibliographic databases (Table 4) and health library services (Table 5) in the provincial, territorial and federal health ministries in Canada.

**Table 2:** Availability of 48 scientific journals for the 14 Canadian health ministries

**Type of access provided**^**a**^	**Number of journals (n = 48)**													
	**Pro**^**b**^										**Ter**^**c**^			**Fed**^**d**^
	**BC**	**AB**	**SK**	**MB**	**ON**	**QC**	**NB**	**NS**	**PE**	**NL**	**YT**	**NT**	**NU**	**HC**
A-Online	21	20	19	0	18	14	6	14	5	18	ND^e^	0	1	34
B-Print-only	2	0	0	0	0	7	2	0	1	3	ND	4	0	0
C-ILL	25	28	29	48	0	27	40	34	42	27	ND^e^	44	47	0
D-‘No access’	0	0	0	0	30	0	0	0	0	0	ND	0	0	14
Total (A + B)	23	20	19	0	18	21	8	14	6	21	ND	4	1	34
Total (C + D)	25	28	29	48	30	27	40	34	42	27	ND	44	47	14
Mean Pro (A + B)	15.0 ± 7.8 (31%)													
Mean Pro (C + D)	33.0 ± 7.8 (69%)													

^a^Online, Access provided through an online subscription (a printed copy of the journal may also be available at the department library); Print-only, Access provided through a print-only subscription; ILL, Access provided through interlibrary loan; ‘No access’, No access provided by any of the previous means (i.e., online subscription, print-only subscription or interlibrary loan).

^b^Provincial health ministries (Pro): AB, Alberta Health and Wellness; BC, British Columbia Ministry of Health Services; MB, Manitoba Health; NB, New Brunswick Department of Health; NL, Newfoundland and Labrador Department of Health and Community Services; NS, Nova Scotia Department of Health and Wellness; ON, Ontario Ministry of Health and Long-Term Care; PE, Prince Edward Island Department of Health and Wellness; QC, Quebec Ministry of Health and Social Services; SK, Saskatchewan Ministry of Health.

^c^Territorial health ministries (Ter): NT, Northwest Territories Department of Health and Social Services; NU, Nunavut Department of Health and Social Services; YT, Yukon Department of Health and Social Services.

^d^Federal health ministry (Fed): HC, Health Canada.

^e^ND, No Data.

**Table 3:** Availability of scientific journals for the provincial and federal health ministries analysed by subject category

**JCR edition (year)**	**Subject category (Number of journals per category)**	**Number of provincial ministries**^**a**^	**Number of journals**^**b**^	
			**Provincial**	**Federal**
Science (2009)	- Health Care Sciences & Services (3)	4.3 ± 1.5	1.3 ± 1.2	2
	- Medical Ethics (5)	3.4 ± 2.5	1.7 ± 1.3	2
	- Medical Informatics (4)	1.5 ± 1.7	0.6 ± 0.8	3
	- Medicine, General & Internal (4)	7.5 ± 0.6	3.0 ± 1.5	4
		(7.7 ± 0.8)^c^	(4.6 ± 1.9)^c^	(6)^c^
	- Medicine, Legal (5)	0.2 ± 0.4	0.1 ± 0.3	5
	- Medicine, Research & Experimental (5)	1.4 ± 2.1	0.7 ± 0.8	4
	- Nursing (5)	1.4 ± 0.9	0.7 ± 1.1	0
	- Public, Environmental & Occupational Health (3)	4.0 ± 1.7	1.2 ± 1.3	3
Social sciences (2009)	- Health Policy & Services (5)	3.0 ± 2.2	1.5 ± 1.5	3
		(3.5 ± 2.3)^d^	(2.1 ± 1.8)^d^	(4)^d^
	- Public, Environmental & Occupational Health (5)	5.4 ± 2.5	2.7 ± 1.6	5
		(5.8 ± 2.5)^e^	(3.5 ± 1.9)^e^	(6)^e^
	- Social Sciences, Biomedical (5)	1.2 ± 0.8	0.6 ± 1.1	2
Mean		3.0 ± 0.8	1.3 ± 0.4	3.0 ± 1.5

^a^Combined data for online and print-only subscriptions. Number of provincial ministries is the mean for three to five journals ± standard deviation in each category. Data for OA journals were excluded for analysis. Data of duplicated journals in two categories were included for analysis.

^b^Combined data for online and print-only subscriptions. For the provincial ministries, number of journals is the mean for ten ministries ± standard deviation in each category. Data of duplicated journals in two categories were included for analysis.

^c^Data for *Canadian Medical Association Journal *and *Cochrane Database of Systematic Reviews *were added to the data in this category for analysis.

^d^Data for *Healthcare Policy *were added to the data in this category for analysis.

^e^Data for *Canadian Journal of Public Health *were added to the data in this category for analysis.

**Table 4:** List of bibliographic databases available to the 14 Canadian health ministries

**Ministry of Health**	**Bibliographic databases**
British Columbia Ministry of Health Services	EbscoHost: Academic Search Premier (ASP), AgeLine, Bibliography of Native North Americans, Biomedical Reference Collection, CINAHL + Full-text, ERIC, GreenFILE, Health Business Elite, Images from Ebsco, Medline with Full Text, PsycARTICLES, PsycEXTRA, PsycINFO, SocIndex, e-Books. OVID: Evidence-Based Medicine Reviews (Cochrane reviews), LWW Journal Collection, Medline. Other: BMJ Clinical Evidence, Canadian Health Research Collection, e-Therapeutics (including CPS), Pharmacist’s Letter/Prescriber’s Letter, Canadian ed., Quickscribe, QP LegalEze, RefWorks citation manager, Science of Early Childhood Development
Alberta Health and Wellness	Health Knowledge Network: PsychINFO, Cochrane Database of Systematic Reviews. EBSCO Databases: Academic Search Premier, Medline, CINAHL Plus with Full Text, Health Business Elite, Psychology and Behavioral Sciences Collection, Health Source: Nursing/Academic Edition, Health Source - Consumer Edition, Biomedical Reference Collection: Comprehensive, Alt HealthWatch, Canadian Reference Centre, Masterfile Premier, Business Source Elite, Canadian Literary Centre, Education Research Complete, Environment Complete, ERIC, Regional Business News, Library Information Science & Technology Abstracts
Saskatchewan Ministry of Health	Saskatchewan Health Information Resources Partnership: Allied & Complementary Medicine Database, Canadian Newsstand, CINAHL Plus with Full Text, e-Therapeutics+, Health and Psychosocial Instruments, Health and Wellness Resource Centre, Health Reference Centre Academic, Health Source: Consumer Edition, Health Source: Nursing/Academic Edition, International Pharmaceutical Abstracts, Natural & Alternative Treatments, Natural Medicines Comprehensive Database, Natural Standard, PsycINFO, BMJ Clinical Evidence, Cochrane Library, Rehabilitation Reference Centre
Manitoba Health	None, but health research support is provided by Manitoba’s Health Information and Knowledge Network.
Ontario Ministry of Health and Long-Term Care	AgeLine, CINAHL with Full Text, InfoTrac General Science eCollection, MEDLINE with Full Text, Academic OneFile, Environmental Studies and Policy Collection, Expanded Academic ASAP, General OneFile, Health & Wellness Resource Centre, The Cochrane Library
Quebec Ministry of Health and Social Services	None
New Brunswick Department of Health	None, but there is a provincial license for Cochrane in New Brunswick, so they do potentially have access to The Cochrane Library
Nova Scotia Department of Health and Wellness	EBSCO Databases: Health Business Fulltext, Health Policy Reference Centre, Medline with Fulltext, Nursing & Allied Health Basic Collection, Psychology & Behavioral Sciences Collection.
Prince Edward Island Department of Health and Wellness	CINAHL, UpToDate
Newfoundland and Labrador Department of Health and Community Services	Newfoundland and Labrador Health Knowledge Information Network: CINAHL Full Text, Biomedical Reference Collection, Cochrane Library, MD Consult, PsycINFO, Psychology & Behavioral Sciences Collection, Social Services Abstracts, Health Business Elite, STAT!Ref
Yukon Department of Health and Social Services	No data
Northwest Territories Department of Health and Social Services	None. Access to Dialog is available through the government legislative library
Nunavut Department of Health and Social Services	None, but health research support is provided by the Neil John McLean Health Sciences Library (University of Manitoba)
Health Canada	AARP AgeLine Database, Agricola, Bibliography of Native North Americans, Canadian NewsStand, CBCA: Canadian Business and Current Affairs, CINAHL: Cumulated Index to Nursing and Allied Health Literature, Cochrane Library, Current Index to Statistics, Econlit [Economic literature], EMBASE, FSTA: Food Science and Technology Abstracts, Global Health, International Pharmaceutical Abstracts (IPA), MEDLINE, MSDS, Newscan, PAIS International: Public Affairs Information Service, Plant Management Network, PsycINFO/PsycARTICLES (full-text), RTECS, Scopus: abstract and citation database, Social Policy and Practice, Social Services Abstracts, Sociological Abstracts

**Table 5:** Health library services in the 14 Canadian health ministries

**Ministry of health**	**Health library services**	
	**Inside**	**Outside**^**a**^
British Columbia Ministry of Health Services	British Columbia Ministry of Health Services, Health & Human Services Library	- Electronic Health Library of British Columbia (e-HLbc)
		- DOCLINE (online interlibrary loan routing and messaging system for health sciences information administrated in Canada by the NRC-CISTI)
Alberta Health and Wellness	Alberta Government Library, Telus Plaza North Tower Site	-Alberta Health Knowledge Network (HKN)
		-NEOS Library Consortium
Saskatchewan Ministry of Health	Saskatchewan Health Library (virtual library environment managed by the Strategy and Innovation branch)	Saskatchewan Health Information Resource Partnership (SHIRP)
Manitoba Health	None^b^	Manitoba’s Health Information and Knowledge Network (MHIKNET)
Ontario Ministry of Health and Long-Term Care	Ontario Ministry of Health Library (virtual library environment managed by the Knowledge Management branch)	None
Quebec Ministry of Health and Social Services	Quebec Ministry of Health and Social Services Resource Centre^c^	-The Digital Network of Quebec Government Libraries^d^
		-DOCLINE
New Brunswick Department of Health	New Brunswick Department of Health Library	DOCLINE
Nova Scotia Department of Health and Wellness	Nova Scotia Department of Health Library	Atlantic Health Knowledge Partnership (AHKP)
Prince Edward Island Department of Health and Wellness	Queen Elizabeth Hospital, Frank J. MacDonald Library	-PEI Government Services Library
		-Maritime Health Libraries Association
		-Support from the University of Prince Edward Island and Dalhousie University (Nova Scotia)
		-Atlantic Scholarly Information Network - Document Delivery Group (ASIN-DDG)
Newfoundland and Labrador Department of Health and Community Services	Newfoundland and Labrador Department of Health and Community Services Resource Centre	-Newfoundland & Labrador Health Knowledge Information Network (NLHKIN)
		-Atlantic Health Knowledge Partnership (AHKP)
Yukon Department of Health and Social Services	None	None
Northwest Territories Department of Health and Social Services	None	Legislative Library of the Northwest Territories
Nunavut Department of Health and Social Services	None	-Nunavut Legislative Library & Information Services
		-Health research support is provided by the Neil John McLean Health Sciences Library at University of Manitoba
Health Canada	Health Canada Library	The National Research Council, Canada Institute for Scientific and Technical Information (NRC-CISTI)

^a^Partnership and/or resource sharing network with other libraries.

^b^On-site support is provided two days a week by a librarian from the Neil John Maclean Health Sciences Library (University of Manitoba).

^c^“Ministère de la Santé et des Services sociaux du Québec, Service des ressources documentaires” in French.

^d^“Réseau Informatisé des bibliothèques gouvernementales du Québec (RIBG)” in French.

Data collected for the five OA journals of our sample, (i.e. *Health Technology Assessment*, *Journal of Medical Internet Research*, *British Medical Journal*, *WHO Technical Report Series*, and *Environmental Health Perspectives*) were excluded from the analysis in Tables 2 and 3, because they provided online access to all research articles without the need of a subscription, which resulted in unexpected data (i.e. ‘no provision’ or ‘interlibrary loan’) for some ministries.

## Results

### Availability of scientific journals

Our first objective was to assess availability of the current issue of a purposive sample of high impact factor journals for Canadian health ministries. We found that the majority of Canadian health ministries did not have subscription access to key journals and relied heavily on interlibrary loans (Table 2). Overall, based on a sample of high-ranking scientific journals (n = 48), availability of journals through interlibrary loans, online and print-only subscriptions was estimated at 63%, 28% and 3%, respectively.

The provincial health ministries provided access to our journal sample primarily by using interlibrary loans (30 ± 13 out of 48 journals; mean for 10 ministries ± standard deviation). Seven provincial health ministries had a similar or higher number of journal subscriptions than that of the provincial ministries average (15.0 ± 7.8 out of 48 journals), whereas three provincial health ministries (i.e. Manitoba Health, New Brunswick Department of Health and Prince Edward Island Department of Health and Wellness) had a ≥2-fold lower number of journal subscriptions than that of the provincial ministries average. For unknown reasons that were not investigated in this study, Manitoba Health was the only provincial ministry that had no department library and relied exclusively on interlibrary loans to provide access to scientific journals, while the Ontario Ministry of Health and Long-Term Care had no interlibrary loan services.

The territorial health ministries of the Northwest Territories and Nunavut had few journal subscriptions (i.e. ≤4, online and print-only subscriptions combined) and relied almost exclusively on interlibrary loan services to provide access to scientific journals. No data were collected for the Yukon Department of Health and Social Services, because of the absence of a department library or a central place that coordinates journal subscriptions. The federal health ministry, Health Canada, used exclusively online subscriptions and provided access to most of our journal sample (34 out of 48 journals). Health Canada had no interlibrary loan services.

To determine whether the provincial health ministries have more subscriptions in one category of journals over another, we analysed the combined results of online and print-only subscriptions by subject category (Table 3). Overall, the number of provincial ministries with a journal subscription by subject category was 3.0 ± 0.8 out of 10 ministries (mean for eleven categories, three to five journals per category ± standard deviation). Four subject categories in the JCR Science Edition (i.e. “Health Care Sciences & Services”, “Medical Ethics”, “Medicine, General & Internal” and “Public, Environmental & Occupational Health”) and one subject category in the JCR Social Science Edition (i.e. “Public, Environmental & Occupational Health”) were above the provincial average. Journals from the “Medicine, General & Internal” subject category had the highest number of provincial ministries with a journal subscription (7.5 ± 0.6 out of 10 ministries), whereas journals from the “Medicine, Legal” subject category had the lowest number of ministries with a journal subscription (0.2 ± 0.4 out of 10 provincial ministries).

To determine whether Health Canada and the provincial ministries have similar subscription patterns for our sample of journals, we analysed the combined number of online and print-only subscriptions by subject category (Table 3). Overall, the number of journal subscriptions by subject category in Health Canada was 2.3-fold higher than that of the provincial ministries average (3.0 ± 1.5 and 1.3 ± 0.4 journals, respectively. Mean for eleven categories, three to five journals per category ± standard deviation). High number of journal subscriptions in Health Canada (i.e. >2.3-fold that of the provincial ministries average) was found for the “Medical Informatics”, “Medicine, Legal”, “Medicine, Research & Experimental”, “Public, Environmental & Occupational Health” (i.e. JCR Science Edition) and “Social Sciences, Biomedical” subject categories. For the federal and provincial ministries, the number of journal subscriptions was highest for the “Medicine, General & Internal” subject category (≥3.0 journals out of 4) and lowest for “Nursing” subject category (<1 journal out of 5).

### Availability of bibliographic databases

Our second objective was to assess availability of bibliographic databases for Canadian health ministries. Indeed, to conduct searches on a regular basis, health ministry staff need full access to bibliographic databases. Free databases can be accessed on the Internet (e.g. MEDLINE, DARE or HSE), however the major discipline-specific (e.g. CINAHL, EMBASE, UpToDate, Cochrane Library or PsycINFO) and multidisciplinary (e.g. Academic Search or Scopus) databases require a paid subscription for full access. Table 4 shows that the federal and provincial health ministries provided access to numerous discipline-specific and multidisciplinary databases, except for four ministries (i.e. Prince Edward Island Department of Health and Wellness, Manitoba Health, New Brunswick Department of Health and Quebec Ministry of Health and Social Services). These latter ministries provided limited or no access to any databases that required a paid subscription. However, Manitoba Health had access to health research support provided by Manitoba’s Health Information and Knowledge Network. The Northwest Territories and Nunavut health ministries did not provide access to databases that required a paid subscription. However, we found that employees of the Nunavut Department of Health and Social Services had access to health research support provided by the Neil John McLean Health Sciences Library (University of Manitoba). No data were available for the Yukon Department of Health and Social Services.

### Health library services

Our last objective was to assess health library services available in the Canadian health ministries. A search of the organisation websites followed by informal email and/or telephonic conversations with government library employees revealed that most (9 out of 10) provincial health ministries had a professionally led library environment, which provides reference services and client support, within their organisation infrastructures (Table 5). Manitoba Health relied exclusively on external health library services. A virtual-only library environment was found within the Ontario Ministry of Health and Long-Term Care and the Saskatchewan Ministry of Health infrastructures. Table 5 shows that provincial health ministries with a department library infrastructure also had access to external health library services available through various library partnerships or consortia, except for the Ontario Ministry of Health and Long-Term Care.

The three territorial health ministries had no department library. Access to scientific journals in the Northwest Territories and the Nunavut health ministries was provided by the legislative library. In the Yukon Department of Health and Social Services, we were not able to collect data concerning availability of peer-reviewed research, because there was no central place that coordinates the journal subscriptions at the time of the study. It is however possible that individual branches in this ministry managed a subscription for a particular journal.

Finally, as for most provincial health ministries, access to a professionally led library environment was found within Health Canada. However, the following library services were provided by the National Research Council, Canada Institute for Scientific and Technical Information (NRC-CISTI): licensing and acquisitions, catalogue, library web site, help desk, cataloguing, and information delivery.

## Discussion

### Summary of research findings

In this study, we investigated availability of scientific journals, bibliographic databases and health library services in the fourteen Canadian health ministries. On the one hand, we found several similarities in availability of peer-reviewed research among the ministries. Firstly, using a sample of 48 scientific journals for analysis, we found that interlibrary loans were primarily used by most health ministries to provide access to scientific journals when compared to online and print-only subscriptions (i.e. 63%, 28% and 3%, respectively). Moreover, online subscriptions (with or without an additional print subscription) were primarily used to provide access to scientific journals when compared to print-only subscriptions (except for the Northwest Territories Department of Health and Social Services; no data were available for the Yukon Department of Health and Social Services). Secondly, most of the organisations provided access to numerous discipline-specific and multidisciplinary databases (except the Prince Edward Island, Manitoba, New Brunswick and Quebec health ministries). Thirdly, a professionally led health library environment was generally found on site (except for the Manitoba, Northwest Territories, Nunavut and Yukon health ministries). Lastly, external library services were generally available together with the department library service through various partnerships or consortia (except for the Ontario Ministry of Health and Long-Term Care).

On the other hand, we found several differences in availability of peer-reviewed research among the ministries. Firstly, five out of the fourteen Canadian health ministries (i.e. the Manitoba, New Brunswick, Prince Edward Island, Northwest Territories and Nunavut health ministries) had a ≥2-fold lower number of journal subscriptions than that of the provincial ministries’ average (15.0 ± 7.8). Secondly, among the provincial ministries, the health ministries in the Maritimes (i.e. New Brunswick, Nova Scotia and Prince Edward Island) had the lowest number of journal subscriptions. Thirdly, Health Canada had a 2.3-fold higher number of journal subscriptions than that of the provincial ministries average in a context where responsibilities for the formulation and implementation of most health care policies and services are almost exclusively in the hands of the provinces and territories. Lastly, for unknown reasons that were not investigated in this study, Health Canada and the Ontario Ministry of Health and Long-Term Care are the only organisations that had no interlibrary loan services (no data were available for the Yukon Department of Health and Social Services).

### Availability of peer-reviewed research evidence in Canadian health ministries

Overall, these data suggest that immediate access (i.e. availability of the current issue through online or print-only subscription) to popular scientific journals in the field of health is limited for six out of fourteen health ministries in Canada (i.e. the Manitoba, New Brunswick, Prince Edward Island, Northwest Territories, Nunavut and Yukon health ministries) when compared to the average number of journal subscriptions available for the provincial health ministries. Therefore, we suggest that the current availability of peer-reviewed research is not optimal and potentially a barrier to health-system policy makers’ use of evidence in several Canadian health ministries. Also, these findings suggest that the use of interlibrary loan as the primary type of provision of scientific journals could be a direct consequence of budget constraints and/or local use. It is therefore not surprising that several health ministries are members of library consortia (Table 5). More research is needed to determine whether lack of subscriptions to the high-ranked journals tested in this study is mediated by provision of lower-ranked journals based on cost-effectiveness analysis (i.e. analyses that include local access, usage - locally collected citation score - and subscription rates). Also, plausible explanations for the discrepancies observed between these organisations may be provided by the assessment of the current supports that Canadian health ministries have to facilitate evidence-informed decision making (e.g. funding, programs, interventions, tools, etc.).

Timely access to high quality research evidence is of the essence in evidence-informed health policymaking [29,45]. Previous studies have shown that policymakers and stakeholders need systematic reviews (SRs) of research evidence to inform policymaking processes [10,11,46]. Here, 33 out of 48 journals (69%) included in data analysis contain SRs in current and/or past issues (Table 1). Journals that had a high number of subscriptions in the provincial and federal health ministries (i.e. ≥8 out of 11 ministries) were high impact factor and/or Canadian journals that address health policy issues, among other topics. Most notable were the journals from the “Medicine, General & Internal” subject category (i.e. *New England Journal of Medicine*, *Lancet*, *Journal of the American Medical Association*, *Annals of Internal Medicine*, *Canadian Medical Association Journal* and *Cochrane Database of Systematic Reviews*). Moreover, using data gathered from Health Systems Evidence (an online repository of syntheses of research evidence about governance, financial and delivery arrangements within health systems, and about implementation strategies that can support change in health systems) [25], we found that *Cochrane Database of Systematic Reviews*, *Journal of the American Medical Association*, *Annals of Internal Medicine, Lancet* and *Canadian Medical Association Journal* were among the top 50 journals that frequently contain SRs of effects or of other questions (i.e. 412, 19, 11, 10 and 5 SRs, respectively) (data not shown). Other journals that match up with our journal sample were also found among the top 50 journals, but had either a low number of subscriptions in the provincial and federal health ministries (i.e., ≤4 out of 11 ministries, for *International Journal of Nursing Studies* - 22 SRs, *Medical Care* - 13 SRs, *Journal of the American Medical Informatics Association* - 8 SRs, *Social Science & Medicine* - 8 SRs, and *Psychiatric Services* - 7 SRs) or were OA (*British Medical Journal* - 40 SRs, and *Health Technology Assessment* - 22 SRs). These findings suggest that, although not optimal, the provincial and federal health ministries provide access to high-quality SRs to inform the policy-making process.

The lack of journal subscriptions by the Canadian health ministries could be mediated by OA journals. Since 2008, Canadian Institutes of Health Research (CIHR) grant recipients have been strongly required to give access to their research outputs in an OA journal and/or in an open Web repository [47]. The importance of OA has also been recognised by other research funding agencies, including the European Research Council [48], the United Kingdom Medical Research Council [49], the United States National Institutes of Health [50], and The Wellcome Trust [51]. However, many journal publishers do not offer OA options for authors yet, or allow only OA to certain articles in an issue for advertising or promotional matters. Indeed, the share of direct (i.e. full) OA articles of all peer reviewed articles indexed by Ulrich’s, Scopus, and Web of Science (ISI) bibliographic databases in 2009 was estimated at 7.7%, 6.8% and 5.9%, respectively [52]. In a recent study, it was shown that the overall access to all scholarly articles through OA publishers and open web repositories was estimated at 8.5% and 11.9%, respectively [53]. Therefore, OA peer-reviewed research remain limited and unlikely to meet the needs of Canadian health ministries within the near future.

Analysis of journal availability in the provincial health ministries showed that online subscription (with or without an additional print subscription) was primarily used to provide access when compared to print-only subscription. A decrease of print journal use with the introduction of online journals was shown in health science libraries during the past decade [54], as the cost per use of print journals can be five times that of online articles accessible by site licenses [55]. Online subscriptions also provide desk access to literature that is likely to be more accessible than in paper-based journals. Although budget constraints for licensing contents and the local use of scientific journals are good arguments in favour of interlibrary loan use in remote locations (such as the territorial health ministries), the loss of productivity resulting from excessive time spent in pursuit of information may be significant for the provincial health ministries. Another impact associated with interlibrary loans use might be the delay for the policymakers requesting the evidence, with the potential for decisions to be made without the available evidence. Extensive cost-benefit analysis would ultimately show why interlibrary loan should be used primarily to provide access when compared to online subscription in the provincial health ministries. Few quantitative studies on the actual use of research evidence by Canadian public health decision makers are available [19,56-58]. The higher (i.e. 2.3-fold) number of journal subscriptions for Health Canada than that of the provincial ministries average could be due to the multi-million dollar initiative of the Government of Canada to provide improved access to information at the desktop for Canadian federal scientists, policy analysts and decision makers [59,60]. To date, all researchers, policy analysts and decision makers in the departments of the Federal Science eLibrary cluster (which includes Health Canada) have access to the entire e-journal collection of Springer, Canadian Science Publishing and the Web Editions collection of the American Chemical Society. Alternatively, adoption of a country-wide approach to provide access to health evidence, such as the Canadian Virtual Health Library [36], would optimise resource expenditures across Canadian health system organisations.

### Study limitations

To the best of our knowledge, this is the first study to systematically address availability of peer-reviewed research in the Canadian health ministries. However, this study has multiple limitations that should be made explicit. Firstly, the cross-sectional nature of the data means that it was not possible to track changes longitudinally. Secondly, collected data are self-reported. Thirdly, our data may not reflect the actual scientific journal availability for all ministry employees, as some of them are pursuing their education and may have privileged access to scientific journals through their learning institutions. Also, availability by pay-per-view was not considered in our study. Fourthly, Thomson Reuters journal impact factors does not reflect a journal’s quality [61], or relevance and applicability to decision-making. Lastly, this study did not require participants to systematically validate their access to any research articles in the current issue of a journal using their Internet access.

## Conclusion

This study provides new insight into availability of scientific journals, databases and library services in the fourteen Canadian health ministries. We found that the majority of ministries did not have subscription access to key journals and relied heavily on interlibrary loans. Study findings have implications for researchers, decision makers and library managers who might wish to improve the availability of peer-reviewed journals within Canadian health ministries, notably those publishing systematic reviews. More research is needed to describe the current access and use of research evidence by policy analysts and decision makers in the Canadian health ministries. A next step could include investigating the correlation between measures of research use and availability of scientific journals, the cost-effectiveness of a different business model for ministerial libraries, librarians’ views about their role (e.g., knowledge brokers and/or custodians of journal collections), and the barriers to availability of scientific journals and databases in health ministries.

## Competing interests

The authors declare that they have no competing interests.

## Authors’ contribution

GL and MO conceived the project and contributed equally to the design of the study, interpretation of the data and writing of the manuscript. All authors read, commented and approved the final manuscript.

## Supplementary Material

Additional file 1 **Availability of scientific journals (n = 53) for the provincial, territorial and federal health ministries in Canada. **Summary of raw data from the survey (Question 1). Click here for file
