# Supplementary material for: Assessing availability of scientific journals, databases, and health library services in Canadian health ministries: a cross-sectional study
Source: Implement Sci. 2013 Mar 21;8:34. doi: 10.1186/1748-5908-8-34 (PMC3616812; doi:10.1186/1748-5908-8-34)
Supplement: Additional file 1 — Availability of scientific journals (n = 53) for the provincial, territorial and federal health ministries in Canada. Summary of raw data from the survey (Question 1). [file 1748-5908-8-34-S1.doc]

**Additional file 1. Availability of scientific journals (n=53) for the provincial, territorial and federal health ministries in Canada.** Summary of raw data from the survey (Question 1).

| **Subject category**  **(JCR Edition)** | **Journal Id^a^** | **Type of access provided^b^** | | | | | | | | | | | | | |
| --- | --- | --- | --- | --- | --- | --- | --- | --- | --- | --- | --- | --- | --- | --- | --- |
|  |  | **Pro^c^** | | | | | | | | | | **Ter^d^** | | | **Fed^e^** |
|  |  | **BC** | **AB** | **SK** | **MB** | **ON** | **QC** | **NB** | **NS** | **PE** | **NL** | **YT** | **NT** | **NU** | **HC** |
| Health Care Sciences & Services  (Science) | 1* | @ | ILL | @ | ILL | @ | ILL | @ | ILL | ILL | ILL | ND^f^ | ILL | ILL | No |
|  | 2* | @ | @ | @ | ILL | @ | ILL | ILL | @ | ILL | ILL | ND | ILL | ILL | @ |
|  | 3 | @ | @ | ILL | ILL | No | Print | ILL | @ | ILL | ILL | ND | ILL | ILL | No |
|  | 4 | @ | @ | ILL | ILL | No | @ | @ | @ | ILL | @ | ND | ILL | ILL | @ |
|  | 5 | @ | ILL | @ | ILL | No | Print | ILL | ILL | ILL | ILL | ND | ILL | ILL | @ |
| Medical Ethics  (Science) | 6 | ILL | ILL | ILL | ILL | No | @ | ILL | ILL | ILL | @ | ND | ILL | ILL | No |
|  | 7 | ILL | ILL | ILL | ILL | No | ILL | ILL | ILL | ILL | @ | ND | ILL | ILL | No |
|  | 8 | @ | @ | @ | ILL | @ | Print | ILL | @ | ILL | @ | ND | ILL | ILL | @ |
|  | 9 | @ | @ | @ | ILL | @ | ILL | ILL | @ | ILL | ILL | ND | ILL | ILL | @ |
|  | 10 | ILL | @ | ILL | ILL | No | Print | ILL | ILL | ILL | ILL | ND | ILL | ILL | No |
| Medical Informatics  (Science) | 11 | ILL | ILL | @ | ILL | No | ILL | ILL | ILL | ILL | ILL | ND | ILL | ILL | @ |
|  | D_2*_ | @ | @ | @ | ILL | @ | ILL | ILL | @ | ILL | ILL | ND | ILL | ILL | @ |
|  | 12 | ILL | ILL | ILL | ILL | No | ILL | ILL | ILL | ILL | ILL | ND | ILL | ILL | @ |
|  | 13 | ILL | ILL | ILL | ILL | @ | ILL | ILL | ILL | ILL | ILL | ND | ILL | ILL | No |
|  | 14 | @ | ILL | @ | ILL | @ | ILL | ILL | @ | ILL | ILL | ND | ILL | ILL | @ |
| Medicine, General & Internal  (Science) | 15 | [@] | @ | @ | ILL | @ | [@] | @ | ILL | [@] | @ | ND | ILL | ILL | @ |
|  | 16 | @ | @ | @ | ILL | @ | Print | @ | ILL | [@] | @ | ND | Print | ILL | @ |
|  | 17 | @ | [@] | @ | ILL | No | [@] | @ | ILL | [@] | @ | ND | ILL | ILL | @ |
|  | 18 | @ | @ | @ | ILL | No | [@] | ILL | @ | [@] | @ | ND | ILL | ILL | @ |
|  | 19* | @ | @ | @ | ILL | @ | [@] | ILL | @ | Print | @ | ND | Print | ILL | @ |
|  | 20 | @ | @ | @ | ILL | @ | [@] | @ | @ | [@] | Print | ND | Print | ILL | @ |
|  | 21 | @ | @ | @ | ILL | @ | ILL | @ | @ | ILL | @ | ND | ILL | [@] | @ |
| Medicine, Legal  (Science) | 22 | ILL | ILL | ILL | ILL | No | ILL | ILL | ILL | ILL | ILL | ND | ILL | ILL | @ |
|  | 23 | ILL | ILL | ILL | ILL | No | ILL | ILL | ILL | ILL | ILL | ND | ILL | ILL | @ |
|  | 24 | ILL | ILL | ILL | ILL | No | ILL | ILL | ILL | ILL | ILL | ND | ILL | ILL | @ |
|  | 25 | ILL | ILL | ILL | ILL | No | ILL | ILL | ILL | ILL | ILL | ND | ILL | ILL | @ |
|  | 26 | ILL | @ | ILL | ILL | No | ILL | ILL | ILL | ILL | ILL | ND | ILL | ILL | @ |
| Medicine, Research & Experimental  (Science) | 27 | ILL | ILL | ILL | ILL | No | ILL | ILL | ILL | ILL | @ | ND | ILL | ILL | No |
|  | 28 | @ | ILL | @ | ILL | @ | ILL | ILL | @ | ILL | @ | ND | ILL | ILL | @ |
|  | 29 | ILL | ILL | @ | ILL | No | ILL | ILL | ILL | ILL | ILL | ND | ILL | ILL | @ |
|  | 30 | ILL | ILL | ILL | ILL | No | ILL | ILL | ILL | ILL | ILL | ND | ILL | ILL | @ |
|  | 31 | ILL | ILL | ILL | ILL | No | ILL | ILL | ILL | ILL | ILL | ND | ILL | ILL | @ |
| Nursing  (Science) | 32 | ILL | ILL | ILL | ILL | No | ILL | ILL | ILL | ILL | @ | ND | ILL | ILL | No |
|  | 33 | ILL | ILL | ILL | ILL | No | ILL | ILL | ILL | ILL | @ | ND | ILL | ILL | No |
|  | 34 | ILL | ILL | ILL | ILL | @ | ILL | ILL | ILL | ILL | ILL | ND | ILL | ILL | No |
|  | 35 | ILL | ILL | ILL | ILL | No | ILL | ILL | ILL | ILL | @ | ND | ILL | ILL | No |
|  | 36 | @ | ILL | @ | ILL | @ | ILL | ILL | ILL | ILL | ILL | ND | ILL | ILL | No |
| Public, Environ-mental & Occupational Health  (Social Science) | 37 | @ | @ | ILL | ILL | No | @ | ILL | ILL | ILL | ILL | ND | ILL | ILL | @ |
|  | 38* | @ | ILL | @ | ILL | @ | ILL | ILL | ILL | ILL | ILL | ND | ILL | ILL | @ |
|  | 39 | @ | @ | ILL | ILL | @ | [@] | ILL | @ | ILL | @ | ND | ILL | ILL | @ |
|  | 40* | @ | @ | @ | ILL | @ | ILL | ILL | @ | ILL | @ | ND | ILL | ILL | @ |
|  | 41 | @ | @ | ILL | ILL | No | @ | ILL | ILL | ILL | ILL | ND | Print | ILL | @ |
| Health Policy & Services  (Social Science) | D_3_ | @ | @ | ILL | ILL | No | Print | ILL | @ | ILL | ILL | ND | ILL | ILL | No |
|  | D_4_ | @ | @ | ILL | ILL | No | @ | @ | @ | ILL | @ | ND | ILL | ILL | @ |
|  | D_5_ | @ | ILL | @ | ILL | No | Print | ILL | ILL | ILL | ILL | ND | ILL | ILL | @ |
|  | 42 | ILL | ILL | ILL | ILL | No | ILL | ILL | ILL | ILL | ILL | ND | ILL | ILL | No |
|  | 43 | @ | ILL | ILL | ILL | No | [@] | ILL | ILL | ILL | ILL | ND | ILL | ILL | @ |
| Public, Environ-mental & Occupational Health  (Social Science) | D_39_ | @ | @ | ILL | ILL | @ | [@] | ILL | @ | ILL | @ | ND | ILL | ILL | @ |
|  | 44 | @ | @ | @ | ILL | @ | [@] | ILL | @ | ILL | @ | ND | Print | ILL | @ |
|  | 45 | Print | @ | @ | ILL | @ | @ | ILL | @ | ILL | ILL | ND | ILL | ILL | @ |
|  | 46 | ILL | ILL | ILL | ILL | No | ILL | Print | ILL | ILL | ILL | ND | ILL | ILL | @ |
|  | 47 | Print | @ | @ | ILL | @ | @ | Print | @ | ILL | ILL | ND | ILL | ILL | @ |
|  | 48 | @ | @ | @ | ILL | @ | Print | ILL | @ | Print | Print | ND | ILL | ILL | @ |
| Social Sciences, Biomedical  (Social Science) | D_6_ | ILL | ILL | ILL | ILL | No | @ | ILL | ILL | ILL | @ | ND | ILL | ILL | No |
|  | 49 | ILL | ILL | ILL | ILL | No | ILL | ILL | ILL | ILL | ILL | ND | ILL | ILL | No |
|  | 50 | ILL | ILL | ILL | ILL | No | ILL | ILL | ILL | ILL | @ | ND | ILL | ILL | @ |
|  | 51 | ILL | ILL | ILL | ILL | @ | Print | ILL | ILL | ILL | ILL | ND | ILL | ILL | @ |
|  | 52 | ILL | ILL | ILL | ILL | No | ILL | ILL | ILL | ILL | @ | ND | ILL | ILL | No |
| NA^g^ | 53 | @ | @ | @ | ILL | @ | @ | ILL | ILL | ILL | Print | ND | ILL | ILL | @ |

^a^Id, Identification number attributed for each journal tested in this study (Table 1). D_Id_, Duplicated journal entry for a designated journal Id. An asterisk (*) indicates an OA journal: All research articles are freely available online.

^b^Type of access provided: @, Online subscription and full access provided online for ministry employees (sometimes, a printed copy of the journal was available at the department library); [@], Online subscription and full access provided online for the department library employees only (access to online research articles was provided on demand by a librarian. Sometimes, a printed copy of the journal was available at the department library); Print, Print-only subscription (access provided using a printed copy of the journal available at the department library); ILL, No subscription by the department library, but the journal was available by interlibrary loan or on the Internet after an embargo period of several months; No, No access provided by any of the previous means (i.e. online subscription, print-only subscription or interlibrary loan).

^c^Provincial health ministries (Pro): AB, Alberta Health and Wellness; BC, British Columbia Ministry of Health Services; MB, Manitoba Health; NB, New Brunswick Department of Health; NL, Newfoundland and Labrador Department of Health and Community Services; NS, Nova Scotia Department of Health and Wellness; ON, Ontario Ministry of Health and Long-Term Care; PE, Prince Edward Island Department of Health and Wellness; QC, Quebec Ministry of Health and Social Services; SK, Saskatchewan Ministry of Health.

^d^Territorial health ministries (Ter): NT, Northwest Territories Department of Health and Social Services; NU, Nunavut Department of Health and Social Services; YT, Yukon Department of Health and Social Services.

^e^Federal health ministry (Fed): HC, Health Canada.

^f^ND, No Data.

^g^NA, Not Applicable.
